# Domain-General Cognitive Skills in Children with Mathematical Difficulties and Dyscalculia: A Systematic Review of the Literature

**DOI:** 10.3390/brainsci12020239

**Published:** 2022-02-10

**Authors:** Francesca Agostini, Pierluigi Zoccolotti, Maria Casagrande

**Affiliations:** 1 Department of Psychology, Sapienza University of Rome, 00185 Rome, Italy; pierluigi.zoccolotti@uniroma1.it; 2 Developmental Dyslexia Lab, IRCCS Fondazione Santa Lucia, 00179 Rome, Italy; 3 Department of Dynamic, Clinical Psychology and Health Studies, Sapienza University of Rome, 00185 Rome, Italy

**Keywords:** mathematical learning disabilities, developmental dyscalculia, mathematical difficulties, executive function, processing speed, working memory

## Abstract

Mathematical performance implies a series of numerical and mathematical skills (both innate and derived from formal training) as well as certain general cognitive abilities that, if inadequate, can have a cascading effect on mathematics learning. These latter skills were the focus of the present systematic review. Method: The reviewing process was conducted according to the PRISMA statement. We included 46 studies comparing school-aged children’s performance with and without math difficulties in the following cognitive domains: processing speed, phonological awareness, short- and long-term memory, executive functions, and attention. Results: The results showed that some general cognitive domains were compromised in children with mathematical difficulties (i.e., executive functions, attention, and processing speed). Conclusions: These cognitive functions should be evaluated during the diagnostic process in order to better understand the child’s profile and propose individually tailored interventions. However, further studies should investigate the role of skills that have been poorly investigated to date (e.g., long-term memory and phonological awareness).

## 1. Introduction

Many studies have highlighted the important role of arithmetic and mathematical skills in everyday life [1,2,3,4], job opportunities and professional success [5,6,7]. However, many school-age children have difficulties learning mathematics, a problem with an incidence ranging between 5% and 7% [8,9,10,11]. Given the clinical relevance of this phenomenon, it is important to understand which factors cause or contribute to mathematical difficulties (MD) in order to intervene more effectively.

Mathematics is a composite discipline, including various domains such as arithmetic, algebra, geometry, and statistics. Individual performance in each of these domains implies developing different skills, such as the sense of numbers, and understanding mathematical concepts and procedures [7,12,13]. Thus, mathematical performance depends on a series of specific domain skills that also require the simultaneous development of general cognitive-domain abilities. The impairment of any of these domains could determine a cascade effect on mathematics learning.

Several studies support the existence of a domain-specific deficit in children with MD [14,15,16] or dyscalculia [17,18]. Butterworth [19,20] proposed that mathematical difficulties in children with Developmental Dyscalculia (DD) are due to a deficit in understanding the basic numerical concepts, such as counting or magnitude comparison (i.e., the number processing system). By contrast, Geary [21] highlighted that competencies in each mathematic domain are based on different conceptual and procedural processes supported by different cognitive abilities. However, the role of such cognitive abilities is still unclear. Indeed, while some studies have shown that domain-general cognitive skills strongly predict mathematical ability [22,23], others reported that mathematical skills depend on both domain-specific and domain-general cognitive processes [7,24,25,26].

Many cognitive functions may be involved in learning mathematics. Processing speed may facilitate carrying out simple tasks, such as decoding numbers and counting quickly, which are useful for speeding up mathematical operations. Furthermore, processing speed is directly proportional to short-term memory storage capacity [27]. Consequently, a higher processing speed will allow the keeping of more information in memory, allowing an association between operations and results [28,29]. The frequent repetition of this process enables information consolidation in long-term memory; this can become an easily and quickly recoverable arithmetic fact [7,30,31], which can increase the automatization of the calculation process. However, phonological awareness may also be important in this process. To solve any calculation, it is first necessary to convert the terms of the operation into a verbal code (i.e., transcoding processes) [29,32,33]. Then, the attentional and inhibitory processes of the central executive system support the procedural and conceptual knowledge underlying each mathematical domain [21,34], as well as the ability to quickly pass from a rule to a procedure or strategy (e.g., shifting, or cognitive flexibility). Furthermore, keeping in memory and manipulating visual and verbal information (e.g., working memory) contributes to mathematical performance [7,21,35,36].

As already highlighted, mathematical domains are numerous, and each of them requires different numerical, conceptual, and procedural knowledge. Different cognitive functions may influence the development of each mathematical skill in different ways. Accordingly, the problems related to the definition and identification of MD are also still open. At a diagnostic level, we refer to math learning disorder (or developmental dyscalculia), when there are persistent difficulties in numerical information processing, memorization of arithmetic formulas, accurate and fluent calculation, with onset in the school years [37]. The diagnosis of dyscalculia is made after a complete and accurate evaluation of specific abilities in numerical cognition (such as subitizing, quantification, seriation, comparison, and calculation strategies) and the procedural level of arithmetic (such as number reading and writing, the ability to perform written operations in the column, and to retrieve arithmetic information and algorithms). However, the instruments currently used to evaluate math skills and identify children with MD do not always evaluate both the formal and “innate” aspects of such skills. This difficulty may lead to incorrect estimates of children’s skill levels [21,38].

A further element of complexity in highlighting MDs and their relationship with cognitive functioning is the use of different classification cut-offs [38] to indicate different severity levels [39].

Moreover, the cognitive domains are usually assessed with “impure” tasks that require a contribution of several processes or abilities [40,41,42]. On the one hand, this implies that the same task may be interpreted as a measure of different domains. For instance, the Stroop task has generally been considered an inhibition task [43], but sometimes it has been considered an attentional one [44,45]. The Rapid Automatized Naming (RAN) task might require various abilities such as processing speed, phonological processing, or visual, temporal, lexical, or attentional processes [46,47]. To deal with this “task impurity” question, we chose to define the cognitive demands assessed by each task according to the authors’ interpretation and theoretical model, except for the RAN. In particular, considering the several abilities involved in this task, we considered RAN as a measure of general processing speed, in line with the idea that naming speed requires rapidly encoding visual stimuli [48,49].

**Figure 1:**
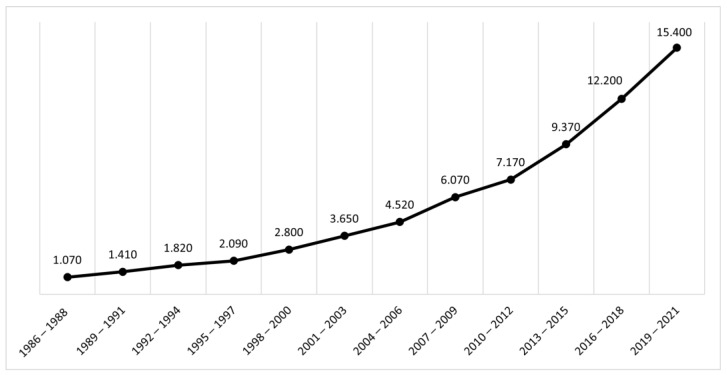
Records on mathematical difficulties and cognitive functions over time (from Google Scholar).

Overall, there has been considerable interest in the relationship between mathematical difficulties and cognitive functions over the last years. An analysis of the number of records on these themes carried out on Google Scholar confirmed this impression (see Figure 1) and indicated a progressively increasing number of papers in this area of research in the last years.

Given all of the above considerations, we set out to carry out a systematic review of this growing body of literature with the aim to:

- Identify the cognitive abilities most impaired in school-age children with MD, independent of the degree of severity and the instruments used for assessment;
- Define which cognitive areas might be helpful to assess to support the potential diagnosis of DD;
- Pinpoint future research areas to enable a more accurate identification of children with mathematical difficulties at risk of DD.

Analyzing the cognitive abilities most impaired in children with mathematical difficulties would allow clinicians to better evaluate the domain-general cognitive skills that influence mathematical abilities, obtaining a more comprehensive profile of their children’s expertise. This knowledge may help clinicians to propose more effective and tuned interventions.

## 2. Method

The review was conducted according to the PRISMA statement [50,51]. The protocol was registered on PROSPERO (CRD42020197079—https://www.crd.york.ac.uk/prospero/display_record.php?RecordID=197079, accessed on 1 October 2020). 

### 2.1. Research Strategies

The systematic search of the international literature was conducted until 20 February 2020, on the following electronic databases: PsycArticles; PsycInfo; Scopus, and Web of Science. The results were limited to articles in English and academic publications. The search was conducted using the following script on each database: ((math* disability OR math* difficulty OR dyscalculia) AND (Cognitive Function*)) and produced a total of 2977 records. After eliminating duplicates *(N* = 273) through the Mendeley software, 2704 records were screened based on title and abstract. Then, 2448 records were excluded, while the remaining 256 were assessed for eligibility based on reading the full texts. 

To update the results, on 8 November 2021, the search was re-run on each database, limiting by publication data range (e.g., 2020–2021). A total of 261 new records was found, and 219 records were screened based on title and abstract after eliminating duplicates (*N* = 42). Finally, thirteen records were assessed based on full texts. 

### 2.2. Eligibility Criteria

Selections were made independently by two researchers, any disagreement between the two judges was dealt with by a supervisor (M.C.). To be included in this systematic review, the studies must meet the following eligibility criteria: (a) school-age participants, i.e., they must be aged between 6 and 12; (b) the study had to evaluate at least one of the following cognitive domains: processing speed, phonological processing; memory (long or short term, verbal and visual, spatial or visuospatial); executive functions such as working memory, inhibition and cognitive flexibility (switching or set-shifting), and attention; (c) the study had to assess the participants’ mathematical abilities and intelligence (e.g., fluid intelligence or verbal or non-verbal IQ).

Cross-sectional and longitudinal studies were included. The cross-sectional studies had to report the measures used for mathematical skill assessment in the screening phase and the criteria adopted to define the group with MD. Furthermore, they had to include a control group. In longitudinal studies, children assessed at preschool-age must have had at least one follow-up during primary school, i.e., during the period of formal math learning. Longitudinal studies also had to include children with persistent MD. Sometimes, MD can be temporarily and spontaneously resolved; therefore, longitudinal studies that did not take this feature into account would not allow us to grasp any cognitive difficulties characterizing the population of interest.

Out of the 269 articles assessed for eligibility, three were excluded because they were in a language other than English, 44 studies were excluded because they were not experimental studies (e.g., book chapters; reviews, metanalyses, theoretical issues, commentaries, or editorials), and 42 were excluded because they did not evaluate the functions of our interest. Furthermore, 10 studies were excluded because they were correlational and, evaluating mathematical skills along a continuum, did not distinguish between children with and without MD. Another ten studies were excluded because they evaluated the neural networks involved in mathematical skills, while five were excluded as they assessed the effectiveness of rehabilitation or enhancement of mathematical skills. Another 65 studies were excluded for methodological reasons (*N* = 32; absence of validated measures for the screening of mathematical skills, classification of the experimental group based on executive and non-mathematical skills), or because they did not involve primary school participants (*N* = 35; preschoolers, adolescents, and adults). Finally, 44 studies were excluded as they assessed the comorbidity between MD and other disorders (*N* = 10), because they did not have a control group without MD (*N* = 6), or because they did not carry out an intelligence assessment of the participants (*N* = 27).

**Figure 2:**
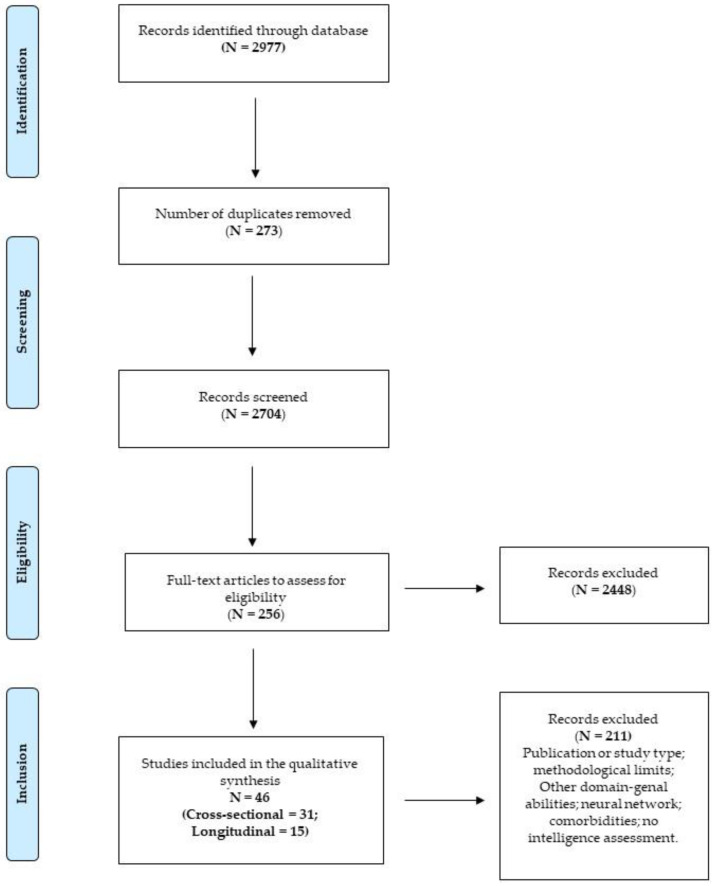
PRISMA flow chart of the selected studies on mathematical difficulties and cognitive functioning.

Forty-six articles were included in the systematic review, 31 cross-sectional and 15 longitudinal studies. Figure 2 reports the flowchart showing the number of studies identified from the databases and the number of studies examined, assessed for eligibility, and included in the review; the reasons for possible exclusions are also reported.

### 2.3. Data Collection and Quality Assessment

The selection of articles was independently conducted by two researchers; a supervisor resolved any doubt. The data of the 46 articles included in this systematic review were extracted according to the PICOS approach [50]. The following information was extrapolated: author(s) and year of publication; study design; characteristics of participants (gender, mean age); tests used to assess intelligence; instruments, and criteria adopted to define the group with MD, cognitive domains assessed and results. Moreover, the number of measurements carried out over time and the cognitive domains (with related instruments) evaluated in the various follow-ups were considered for the longitudinal studies. Table A1 and Table A2 in the Appendix A reports the extracted data for cross-sectional and longitudinal studies, respectively.

The results are summarized, reporting the performance differences between the MD and control group. The quality of the studies was assessed using the Cochrane Handbook for Systematic Reviews criteria [52], adapted ad hoc according to the objective of this review. For each study, the evaluated domains were: (a) selection of the sample and control of any variables that could play a role in mathematical difficulties (e.g., IQ, socioeconomic status, motivation or performance in reading tests; selection bias); (b) the use of standardized instruments to assess mathematical skills and a clear definition of the MD group (selection bias); (c) the use of appropriate tasks or tests for assessment of the cognitive domains considered (detection bias); (d) incomplete outcome data about cognitive functions (attrition bias); (e) selective outcome reporting in the discussion (reporting bias), and (f) other risks of bias. 

The quality of the studies was categorized with an unclear/low/high risk of bias for each item (“0” for a low risk of bias, “1” for a high risk of bias, “Unclear” otherwise). For each study, a mean score was calculated and multiplied by 100. Then, studies were categorized into a low risk of bias (lower than 75%) or a high risk of bias (higher than 75%). Finally, if at least two items were unclear, the study was classified with an unclear risk of bias.

## 3. Results

### 3.1. Studies Selection

The systematic search produced a total of 3196 records. After eliminating duplicates (*N* = 315) and the screening based on title and abstract, 269 articles were evaluated for eligibility, then 46 were included in the qualitative analysis, i.e., 31 cross-sectional and 15 longitudinal studies (see Figure 2).

The studies meeting the inclusion criteria were conducted from 1980 to 2021 and involved 8506 children. Participants were aged between 7 [53,54] and 11 years [44,55,56,57,58]. The percentage of females in the studies ranged from 31.8% [38] to 83.3% [59]. In five studies, information on the participants’ gender was not reported [55,60,61,62,63].

### 3.2. Quality Assessment

**Figure 3:**
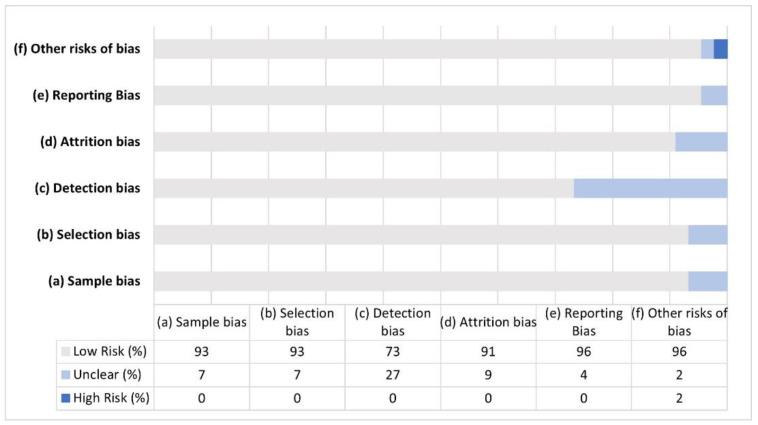
Percentage of risk of bias for each domain of tool assessment for the selected studies.

Figure 3 shows the percentage of articles fulfilling each quality criterion assessed. All but three studies had a generally good quality, with an average risk of bias lower than 75%. The high percentage of studies with low (37.8%) or no risk (55.5%) of bias highlights the validity of this systematic review. No study reports a high risk of bias, while three studies (6.7%) showed an unclear risk of bias. All studies clearly defined the criteria for the MD group and used appropriate statistical analyses. The highest risk of bias was in the detection bias domain (27%), and was due to the assessment of cognitive functions with non-standardized tools that produced some concern of risk of bias.

### 3.3. Characteristics of Selected Studies

The characteristics of selected studies are organized into two subsections: characteristics of cross-sectional and longitudinal studies. 

#### 3.3.1. Characteristics of Cross-Sectional Studies (*N* = 31)

The mean age of children with MD ranged from 7 to 11 years. The most represented age group was 9 years, considered in 12 out of 31 studies (38.7%) [59,64,65,66,67,68,69,70,71,72,73,74]. Two studies did not report the mean age of the sample, but the participants were recruited in primary school classes; therefore, they fall within the age range of our interest [31,75].

Studies included in this review assessed intelligence using different tests, such as Raven’s Standard Progressive Matrices (RSPM) [44,56,57,60], Raven’s Colored Progressive Matrices (CPM) [59,70,71,74,76,77], Cultural Fair Intelligence test (CFT) [72], reduced versions of different editions of the Wechsler Intelligence Scale for Children (WISC-R- [58]; WISC III- [47,53,61,68,69,76,77,78]; WISC IV [73,79]), the Wechsler Abbreviated Scale of Intelligence (WASI) [31,54,64,65], the Stanford–Binet Intelligence Scales [75], the Primary Mental Abilities (PMA) [66,67]; the Intelligence and Developmental Scale (IDS) [80], and the Peabody Picture–Vocabulary test (PPVT) [55].

The instruments used for the initial assessment of mathematical skills, i.e., for defining groups with MD, are reported in the Supplementary Materials (Table S1). Table S1 also includes the mathematical domains assessed and the cut-offs applied to define children with MD.

#### 3.3.2. Characteristics of Longitudinal Studies (*N* = 15)

Each longitudinal study included at least one assessment in the first-grade primary school and the definition of MD according to at least two assessments of math achievement.

The studies assessed intelligence using either the Raven’s Colored Progressive Matrices (CPM) [63,81,82], Raven’s Standard Progressive Matrices (RSPM) [83], Vocabulary and Matrix Reasoning subtests of the Weschler Intelligence Scale for Children (WISC-III) [84,85], the Weschler Abbreviated Scale for Intelligence (WASI) [38,86], the Receptive Vocabulary subtests and the Drawing with Cubes of the WPPSI-III [62]. Other studies used two intelligence measurements, i.e., CPM and some subtests of WISC-III [87] or WASI [88,89,90], at two different points in the study. Finally, a study evaluated verbal IQ through the PMA battery [91], while in the Mazzocco and Grimm study [92], the test used is not specified, but an IQ higher than 80 is reported in the participants. 

The instruments and criteria used to assess mathematical skills and their persistence, i.e., for defining groups with MD, are reported in the Supplementary Materials (Table S1).

### 3.4. Results on Cognitive Functioning (N = 46)

The studies included in this systematic review refer to developmental dyscalculia (*N* = 5), mathematic learning disabilities (*N* = 13), MD (*N* = 19), or mathematical disability (*N* = 9) to consider conditions that appear similar. Notably, the use of these terms was not clearly influenced by the cut-off criteria used to define the severity of mathematical deficit. To report the results of the studies, we chose to refer more generally to mathematical difficulties. In such a way, we included both children who performed well below average (e.g., 10° percentile) and those performing at or below the 35th percentile (e.g., less restrictive criteria). 

The studies that evaluated the difference between groups with and without MD considered the following cognitive domains: processing speed (*N* = 22), short-term (*N* = 13) and long-term memory (*N* = 2), attention (*N* = 9), executive functions such as working memory (*N* = 32), cognitive flexibility (*N* = 7), inhibition (*N* = 8), and phonological awareness (*N* = 4). The results will be separately presented and summarized for each cognitive domain. 

Table 1 summarizes the number of studies reporting worse performance in the MD group than in the control group (CG) for each cognitive domain.

#### 3.4.1. Processing Speed (*N* = 22)

Twenty-two articles evaluated the processing speed of children with MD compared to a control group. Among these, fifteen were cross-sectional studies, while seven were longitudinal studies.

Thirteen studies evaluated the ability to process visual stimuli, and most of them adopted barrage tasks, i.e., visual search tasks [31,54,60,67]. Lafay and St-Pierre [59] used a coding task, while composite scores derived from barrage and coding tests were used in two other studies [58,81]. A lower accuracy in performing these pencil and paper tasks within a time limit was observed in all studies [31,54,59,67] except in Chan and Ho’s study [60]. A worse performance of children with MD was also observed in a task demanding the identification of the number of dots on certain cards [72].

By contrast, no difference emerged in studies using simple reaction time (RTs) tasks [44,93] or choice RT tasks [79] to assess processing speed. 

Fourteen studies evaluated the ability to name stimuli rapidly, i.e., Rapid Automatized Naming (RAN). In this task, participants had to name alphanumeric, such as digit and letters [38,47,54,60,61,81,87,88,89,90,92], or non-alphanumeric stimuli, such as colors or pictures [38,47,61,63,85,92]. Other authors used a composite score obtained from the speed in naming letters, digits, and colors [58]. In studies requiring participants to name colors or pictures quickly, a worse performance was found in children with MD [38,47,61,63,92].

Longitudinal studies using a RAN task with color naming identified an MD group’s persistent slowness even at follow-up [38,92]. Specifically, this difficulty persisted only in children classified according to the 10th percentile [38,92], while children classified with the 25th percentile were slower than the control group only until 6 years of age. One study [85] did not find a worse performance in the MD group than in the typical achievement group. 

Three cross-sectional studies [54,60,61] and six longitudinal studies [81,87,88,89,90,92] used alphanumeric stimuli and found worse performance in children with MD than in the control group. Only Donker and colleagues [47] did not observe any difference between groups in the speed of naming alphanumeric stimuli. 

Furthermore, two studies [60,92] found worse performance in naming digits only in the group of younger children with MD (mean age = 8.3), but not in older ones (10 years; [60]) and at the follow-up (14 years; [92]). Murphy and colleagues [38] reported a persistent slowness in naming digits at all follow-ups (up to the third grade, 8 years) only in children with MD classified in the 10th percentile; conversely, the children classified according to the 25th percentile at the last follow-up (third grade) presented a performance equivalent to that of the control group. The slowness of naming letters and colors found in preschoolers persisted even at the follow-up when the children were ranked in the 10th percentile (in 8th grade [92]).

##### Synthesis of Results and Comments

Processing speed could be assessed with several tasks requiring abilities involved in relatively simple cognitive tasks [94].

This systematic review highlights low processing speed for visual and verbal stimuli in children with MD in most studies (Table 1). Specifically, in visual processing, these difficulties were manifest in the execution of visual search tasks that required the participant to identify the target stimulus among other distractors as quickly as possible [31,54,67] or when the task consisted in reproducing symbols associated with single numbers or letters [81]. The only study that used a composite score (barrage and coding tasks) identified a worse performance in children with MD than in the control group [58]. The same difficulty occurred in the numerosity processing task [72], demanding that participants process the numerosity of the elements represented on a map and quickly calculate the solution. It seems interesting to note how these pencil-and-paper tasks were more sensitive to identifying any difficulties in children with MD (with respect to the control group) than studies using simple RTs as an indicator of processing speed. RTs did not identify differences between children with and without MD [44,79,93], regardless of the cut-off scores and the screening measures used to define the experimental group.

In the Rapid Automatized Naming tasks, children with MD have greater difficulty, mainly linked to a slow execution compared to the control group, regardless of the type of stimulus presented [38,47,54,61,81,87,89,90,92]. However, in Chan and Ho’s study [60], only younger children with MD were slower in naming a series of pictures, while this difference disappeared when the older group was considered. 

In the longitudinal studies, in which the group of participants with MD was classified according to the persistence of the difficulties, a slower performance in processing-speed tasks persisted only in children classified using a cut-off at the 10th percentile [38,92].

#### 3.4.2. Short- and Long-Term Memory (*N* = 12)

Among the 12 studies that evaluated verbal short-term memory, most of them did not report worse performance in the MD group compared to the control group [57,59,67,69,71,76,80,84,91,93,95]. These studies used stimuli words [57,69,71,90], non-words [95], or numbers [57,59,67,69,71,76,80,84,91,93]. In order to verify whether the type of stimulus can influence the performance of children with MD, some studies compared their performance in digit and letter [80] or word span tasks [57,67,69,71,91]. The results did not highlight differences based on the type of stimulus proposed [67,71,80,91]. Other authors [57,69] found a worse performance in children with MD exclusively in digit span tasks and not in word span tasks. Webster [55] confirmed this finding regardless of the nature (visual or verbal) of the stimulus and the type of response (written or verbal). 

Visuospatial short-term memory was evaluated in four studies. Three studies found no difference between the groups with and without MD using the Corsi Block test Forward [59,69,93], while Szucs and colleagues [71] observed worse performance in children with MD using a Dot Matrix task.

Long-term memory was analyzed in its verbal component only in two studies. Reimann and colleagues [80] found no difference in the ability to recall a story among children with and without MD, while children with MD performed worse on a semantic fluency task [31].

##### Synthesis of Results and Comments

Nine out of twelve studies evaluating short-term verbal memory found worse performance in children with MD than the control group in tasks that used numbers, letters, words, or non-words as stimuli (Table 1). Only three studies using the Digit Span Forward did not identify differences between the two groups.

All of the studies that analyzed verbal memory have presented the stimuli requiring a verbal response. Webster’s study [55] assessed whether the performance of children with MD could depend on the modality of presentation and recall of the stimuli, and found that children with MD recalled more elements when they had to reproduce them verbally than in a graphic–symbolic way; the opposite trend occurred in children with adequate mathematical skills [55].

Concerning the short-term visuospatial memory, the Corsi Block task did not highlight differences between children with and without MD [59,69,93].

Long-term memory has been evaluated only in its verbal component. Using a semantic fluency task, the performance was worse in children aged 8 years with MD; this finding did not occur in children of 10 years or older [31]. However, Reimann and colleagues [80] did not find differences between groups requiring children to recall a story after a latency period.

#### 3.4.3. Attention (*N* = 9)

Attention was assessed in nine studies. One of them observed the worst performance in children with MD than the control group in divided attention tasks (e.g., dual-task) that required participants to read a sentence or an operation on the computer screen and remember the last word or the result [57]. 

Children with MD also seem to show greater difficulty in selective attention tasks, in which they were asked to identify elements with a given characteristic, ignoring irrelevant information [80]; this task also implies processing speed. Willcutt and colleagues [58] observed a greater number of omissions in children with MD than in the control group in an 18-minute task in which they were required to press a button when the number “9” appeared immediately after the number “1”. With a similar task using images rather than numbers, Kuhn and colleagues [79] observed only a greater number of false alarms in children with MD than in the control group. Worse performance in attentional tasks also emerged in the Cai and colleagues’ study [44], who assessed this ability through expressive attention (e.g., Stroop task), number detection (e.g., visual search), and receptive attention (e.g., determining whether the letters presented were physically the same or if they have the same name). Finally, four studies evaluated attention through the Strengths and Weaknesses of ADHD and Normal Behavior (SWAN) administration and reported higher scores in the inattention subscale [31,54,65,89] and hyperactivity/impulsivity scale [64] in children with MD, compared to the control group.

##### Synthesis of Results and Comments

Most of the included studies observed a worse performance in attentional tasks (Table 1); children with MD presented difficulties in both vigilance/sustained attention tasks [58,79] and those evaluating selective attention in a limited time [80]. Moreover, higher inattention ratings were detected through SWAN in the MD group. 

#### 3.4.4. Executive Functions

##### Working Memory (*N* = 32)

Twenty-one studies assessed verbal working memory and fourteen assessed visuospatial working memory. Five studies evaluated working memory according to the Baddeley model [96].

Concerning verbal working memory, fourteen studies found significantly lower scores in children with MD, compared to the control group, both in Digit Span Backward tasks [31,44,54,58,59,60,69,70,76,83,84,91,95], and in Word Span Backward tasks [69,79,91]. A significant difference also emerged in the Listening Span task [67] and Sentence Digit task [56] in which the participant was required, respectively, to recall the last word of a sentence pronounced by the experimenter after having given a judgment of its truthfulness, and to recall the number of the street/address pronounced by the investigator. However, controlling for the number of intrusions, any difference in performance disappeared in the Listening Span task [91]. However, the difference persisted in the Listening Span completion task, which required the participant to recall the words he/she used to complete some incomplete sentences. Five studies did not observe a different performance between the two groups using the Listening Span test [71], the Digit Span Backward [82,93], the Word Span Backward [90], or a composite score derived from the number of correct responses in an Auditory Digit Sequence and a Semantic Categorization test. In this task, the participant recalled an address and placed a series of words in the correct semantic category [74].

Out of eight longitudinal studies evaluating working memory, five referred to the Baddeley model [81,87,88,89,90] and identified a worse performance of children with MD compared to the control groups both in the tasks evaluating the phonological loop and in those assessing the central executive. Even in Cai and colleagues’ study [44], in which the central executive was assessed through the Stop-Signal and the Flanker tasks, children with MD performed worse than the control group. Only one study [85] did not find a difference between groups using a task that requires participants to recall, in the correct serial order, a sequence of words while managing an interferential task (e.g., deciding whether each presented word was an animal or not).

Concerning the results in tasks measuring the visuospatial sketchpad, two studies reported a worse performance in children with MD [88,89], while three did not detect any difference [81,87,90] although they used the same tool (WMBT-C).

Out of the 14 cross-sectional studies evaluating visuospatial working memory, nine identified a significant difference between groups with and without MD in visuospatial working memory [44,56,60,69,71,73,74,78,79]. The visuospatial memory tasks proposed to the participants were different. Some used the Mapping and Direction task [56,74], requiring the participant to memorize the symbols found on a path and then recall them. In other studies, different versions of the Visual Matrix task [74,79] or the Spatial Span [69,78] were used, in which the participants were required to recognize in which spaces of a grid (Visual Matrix span, Dot Matrix, Nine-grid task) or a figure (Spatial Span and Odd One Out) the dots were previously shown. In other cases [60,73], the child had to recognize the figures previously shown among distractors, while in the Cai and colleagues’ study [44], the 2-back task was proposed, in which the child must press a button if the figure appearing on the screen was the same as that shown one or two times before.

On the other hand, five studies, using the Corsi Block Recall Backward test [59,69,70,84,93]. Furthermore, one study [80], using a subtest of IDS (recognition of tridimensional figures), did not confirm any difference between children with and without MD. 

##### Inhibition and Interference Control (*N* = 8)

Four studies used the Color–Word Stroop task [4,57,58,77], and only one of them [58] observed a worse performance in children with MD than in the control group.

Worse performance in children with MD emerged using the Number Inhibition task [57,74], while no differences were observed employing the Numerical Stroop task [53].

Poorer performance in children with MD than in the control group was reported with the Stop-Signal task [58,71], which requires participants to inhibit an automatic response (press a button when targets appeared) in the presence of a given stimulus (alert sound). Children with MD also had a higher number of false alarms in the Visual Continuous Performance (CPT) task in which they were required to press a button when a “9” appeared immediately after a “1” [58]. The difficulty of children with MD in cognitive control tasks was also confirmed by Cai and colleagues [44].

The only study using the Go/No-Go task [68] did not find more impaired inhibition in children with MD than the control group. Equally, Censabella and Noël [77] did not find a different performance in children with MD and in the control group in a task requiring suppression of irrelevant information from working memory or performing a Flanker task.

##### Cognitive Flexibility (*N* = 7)

Within the four cross-sectional studies evaluating cognitive flexibility, two [71,74] observed a worse performance in children with MD than the control group in a Trail Making test (TMT).

Willcutt and colleagues [58] found that children with MD made more perseverative errors in the Wisconsin Card-Sorting test (WCST). However, Kuhn and colleagues [79] did not observe any difference between children with and without MD using a PC-based flexibility task.

Three longitudinal studies [38,62,86] adopted composite tasks involving different executive functions (e.g., working memory, inhibition, cognitive flexibility). Specifically, two studies [38,86] used the Contingency Naming test (CNT) that required children to name the stimulus according to one attribute (e.g., color or form, based on the stimulus congruence) or two-attribute (color or form based on the stimulus congruence and the presence/absence of an arrow) rules. In the first assessment (e.g., 1st grade), the MD group defined by a 10° cut-off showed less efficient performance than the control group on the one-attribute subtest [38,86], while the MD group defined by a 25° cut-off did not [38]. Regarding the two-attribute subtest, Murphy and colleagues [38] assessed MD performance only in 4th grade, showing the worst performance in both MD groups (defined by the 10° or 25° percentile), while Mazzocco and Kover [86] did not analyze this subtest, because only one child with MD completed the subtest on the first assessment. 

The worst performance in children with MD was also observed in the Chu and colleagues’ study [62], adopting the Conflict Executive Function Scale, which required participants to place cards inside two boxes based on different rules (congruence or incongruence of the stimuli; color or shape; color or shape based on the presence/absence of the border on the card).

##### Synthesis of Results and Comments

The number of studies finding a different performance between MD and CG groups on each executive function are summarized in Table 1. In general, children with MD presented a critical performance in visuospatial working memory tasks [44,56,60,69,71,73,74,78,79]. Nevertheless, it is interesting that such difficulties emerged in many tasks, but not when the Corsi Block task was used [59,69,70,93]. This finding suggests that this task may not be sensitive in identifying specific difficulties in visuospatial working memory in children with MD. Concerning other executive functions, children with MD were generally impaired in tasks evaluating cognitive flexibility [58,71,74], inhibition of automatic responses, interference control (i.e., the Stroop Task or its “numerical” variants), and attentional control (i.e., the dual-tasks independently from the numerical nature of the stimuli; [57]). Only Kuhn and colleagues [79] did not identify any difference using a Choice Reaction Times task.

#### 3.4.5. Phonological Processing and Phonological Awareness (*N* = 4)

Three cross-sectional studies [54,58,61] and one longitudinal study [63] assessed phonological processing or phonological awareness. 

One study observed worse performance in phonological awareness assessed through a composite score, including both deletion (phoneme deletion from a word or a non-word) and manipulation of phonemes (move the first phoneme of a word to the end and then add a sound [58]). The study of Slot and colleagues [61] found a worse performance of the MD group than the control group when the task required participants to delete the onset, middle, or last sound from a word (e.g., phonemic deletion task). No differences between the groups with and without MD were found in tasks that required switching the first sound of two given words [61], removing a sound (syllables or phonemes) varying in position [54], or identifying the initial phoneme [63].

#### Synthesis of Results and Comments

These few studies, including phonological processing and awareness assessment, indicate mixed results (Table 1). The lack of homogeneity between the tasks proposed does not allow inferences. 

## 4. Discussion

The purpose of this review was to identify the cognitive skills involved in MD. Finding general skill deficits in children with MD would be advantageous in clinical assessment because it could help recognize children with specific mathematic learning disabilities. During the diagnostic process, an assessment of cognitive skills is already recommended [97,98], but there is no agreement on which skills should be of greatest interest.

This review highlights that children with MD have greater difficulties than matched controls in several measures of processing speed, working memory, inhibition, and cognitive flexibility.

In particular, the difficulties related to the processing of visual stimuli are more manifest in tasks requiring visual and perceptual discrimination, such as in visual search tasks [31,44,54,67,72] or coding tasks [44,59]. By contrast, children with MD are no slower than children without MD in responding to visual stimuli, as shown by studies using simple reaction time tasks to assess processing speed [44,79,93]. Therefore, their impairment would not depend on the ability to respond promptly to a stimulus, but rather on the request to process this stimulus and recognize its relevant characteristics quickly. This cognitive aspect would also imply the ability to discriminate stimuli correctly. From this point of view, a deficit in visual processing would entail difficulty discriminating between numbers and arithmetic signs [99,100]. These difficulties would affect formal mathematic learning [101,102].

Conversely, children with MD do not appear to have verbal and visuospatial short-term memory difficulties. However, the results concerning verbal short-term memory seem to contrast with the findings of some recent reviews, in which a worse performance of children with MD has been identified by using these tasks [29,103]. Moreover, according to some authors, children with MD would especially have difficulty in memorizing numerical information. In the studies included in this review, only two out of the eleven studies evaluating performance in verbal and numerical span tasks identify this trend in children with MD [57,69].

The results on verbal working memory are in line with previous reviews [29], showing worse performance in children with MD [31,44,54,56,58,59,60,67,69,70,76,79,83,84,91,95]. Similarly, visuospatial working memory is impaired in children with MD [44,56,60,69,71,73,74,78,79], in line with a previous meta-analysis on this topic [104].

The limited working memory capacity of children with MD, linked to normal short-term memory, could indicate a specific difficulty in retaining information and simultaneously performing manipulations or operations [76]. This difficulty would not emerge in tasks in which the cognitive load is lower, as in the direct memory span task, which requires passive repetition of elements [103].

Another interesting finding is the impairment in attentional control [57] and sustained attention over time [58,79] in children with MD. The difficulties in tasks requiring both verbal and visual manipulation and involving attentional processes could explain the high comorbidity between attention deficit hyperactivity disorder (ADHD) and MD [105,106,107].

Deficits in working memory and attentional control could affect mathematical performance, especially in those tasks that require multistep planning and processing of information, as occurs in the case of MD [29,108].

Children with MD present normal inhibition abilities when assessed through the classic Stroop task [57,71,74,77] or the Stroop task using numbers [53]. However, they show greater difficulties in solving the Number Inhibition tasks [57,74]; in fact, they ignore the presented number, indicating only the quantity of digits (e.g., in the presence of the stimulus “444”, they may say: three, referring to the number of digits rather to the quantity indicated by the number). An inhibition difficulty also emerged in the Stop-Signal task that requires participants to inhibit a response (press a button) previously made automatically [44,58,71]. This impairment appears evident in some typical errors that children with MD commit in retrieving arithmetic tables [109,110]. However, the inhibition could also be linked to suppressing ineffective strategies in favor of new, more efficient strategies, revealing high cognitive flexibility [111]. In fact, children with MD have more difficulty changing their response based on the demands of the context [38,58,62,71,74], and this difficulty could affect children’s ability to perform complex mathematical calculations in which it is necessary to go from one procedure (e.g., subtraction) to another (e.g., multiplication).

Furthermore, slowness in Rapid-Naming tasks appears to be a common feature in children with MD [47,54,58,60], as it shares with arithmetic some basic processes, such as the rapid retrieval of phonological representations from long-term memory [112]. However, when compared to alphanumeric RAN, the results appear to be mixed. According to Donker and colleagues [47], alphanumeric RAN would mainly involve phonological processing ability, but this skill was not examined in depth in their study. The only study that evaluated phonological processing and Rapid Naming [58] observed worse performance of children with MD in both tasks, supporting Donker’s hypothesis.

In non-alphanumeric RAN, children with MD showed worse performance, presumably because it involves elements related to the conceptual and perceptual processing of objects and the ability to use the verbal and visual code interactively [47,113]. Furthermore, it involves the retrieval of semantic information [114]. Therefore, non-alphanumeric RAN requires additional processes compared to alphanumeric RAN, in which children with MD could be specifically and uniquely compromised [47]. On the other hand, other authors [115] found that visual processes, such as visual discrimination, visual problem-solving (e.g., reasoning tasks with matrices), and attention are central in non-alphanumeric RAN tasks.

Words have both a semantic and a phonological representation in the mental lexicon. Letters have phonological representations, but they do not have a meaning [116]. Therefore, the naming of letters activates mainly phonological access; conversely, naming an image requires semantic access. Phonological and semantic accesses are two separate mechanisms that could independently contribute to mathematical skills. Specifically, the difficulty in non-alphanumeric RAN tasks could reflect difficulty integrating the visual-perceptive information (the image of the stimulus) with its semantic representation. It could also be interesting to verify whether children with MD have a specific difficulty in the RAN of numbers, reflecting the same difficulty of symbolization present in the RAN of pictures.

Consequently, the core deficit in children with MD could be linked to visual–perceptual discrimination and rapid scanning of visual information; this impairment would undoubtedly explain the difficulties in visuospatial working memory and perhaps those in verbal working memory.

This finding, combined with those concerning visuospatial skills, would support the hypothesis of a “visual system” [21] necessary to organize and manipulate the information involved in the procedural knowledge that allows good math performance.

## 5. Limitations

Some cautions must be considered when interpreting the results of the present review.

First, some limitations concern the definition of the mathematical deficit itself. On the one hand, there is no agreement on which term better describes this condition [117,118]. On the other hand, the standardized measures to assess math achievement do not evaluate all the numerical, mathematical, and arithmetic domains that could be compromised in specific learning disabilities in mathematics. Moreover, there is no full consensus about the cut-off criteria for MD or DD diagnoses. Consequently, different cut-off points and various standardized tests are used across studies. These different methodological approaches might determine the presence of heterogeneous samples. By including only studies that assess intelligence we aimed to control for the potential effect of low intellectual ability on mathematical skills. This precaution made it possible to exclude that the poor performances in mathematics could depend on a general intellectual deficit. 

Another aspect that must be considered concerns the potential role of factors, such as language, that appears to be predictive of mathematical learning [119,120]. Most of the studies analyzed in this work did not evaluate linguistic skills and did not define the MD group based on both linguistic and mathematical performance. For this reason, we could not analyze the potential role of linguistic skills on MD, and this remains to be carried out in future research.

Another important aspect to consider is the high variability of the tests used to evaluate the different cognitive domains, which prevented a meta-analysis of the results. Consequently, a limitation of this review is the lack of a quantitative analysis that would have given greater force to the inferences through effect-size analyses. In fact, the qualitative analysis does not consider the power of rejection of the null hypothesis; therefore, it is impossible to distinguish studies with different rejection power. The tests used to evaluate cognitive functions certainly represent a crucial problem because they include a pool of many different aspects. For example, if we consider attention, it refers to a multi-component system (see Petersen and Posner’s model; [121]), including qualitatively differentiated aspects of attention, such as social attention (e.g., [122]) that presents a critical performance in other developmental disorders (e.g., [123]). Furthermore, mathematical difficulties could be associated with critical interactions between attentional systems rather than with an impairment of a single attentional system (e.g., vigilance, selective attention, etc.). Therefore, it would be useful to use tasks such as the Attentional Network test (ANT; in the versions suitable for children, e.g., [124,125,126]), which can simultaneously assess the efficiency of attentional systems and their interactions. This suggestion is supported by the fact that the ANT is also sensitive to highlighting attentional deficits in other neuropsychological disorders (e.g., [127]).

One additional limit could be publication bias. Some methodological choices allowed the definition of rigorous inclusion criteria for the studies, which lead to not analyzing any grey literature. The choice to include only academic articles published in peer-review journals may have limited the selection only of those studies that have obtained results in line with the literature. 

## 6. Conclusions

Mathematical performance implies a series of numerical and mathematical skills that are strictly linked to some general cognitive abilities that, if impaired, may have a cascading effect on math learning. This systematic review aimed to identify the most impaired cognitive functions in school-age children with MD. Despite some variability in the tests used to evaluate the various cognitive domains, the main findings revealed poor executive function performance, such as inhibition, flexibility, working memory, and processing speed. These domains should be assessed when a disorder is suspected, in order to support the potential diagnosis and provide an ad hoc treatment.

Furthermore, this review highlights the need to develop a standardized protocol to assess this specific mathematical learning disability. This protocol should consider many mathematical skills representing this complex domain, including formal and informal competencies and general domain abilities such as working memory, processing speed, and executive functions. Mathematical difficulties can be specific to a mathematical domain or represent the outcome of difficulties in other cognitive domains. Defining these domains can make it possible to verify whether mathematical difficulties result from a specific cognitive deficit (e.g., working memory, attention, etc.). In this case, it would be possible to distinguish children with specific deficits in the mathematical area and children for whom mathematical difficulties depend on deficits in other cognitive domains. In the first case, rehabilitation interventions could directly address mathematical skills; in the second case, they might at least initially address the cognitive domain impaired.

## 7. Open Challenges and Research Directions

The present work has shed some light on the cognitive abilities that are most impaired in children with mathematical difficulties. Still, some critical issues remain open. First, some cognitive domains, such as long-term memory and phonological awareness, have received scant attention; further studies are needed to understand their role in mathematical learning and difficulties. Second, a future systematic review could investigate the role of language on different mathematical domains, to better understand how linguistic skills support each mathematical domain. Finally, some cognitive abilities appear to be compromised independently of the severity of the mathematical difficulties, suggesting that these competencies could support mathematical learning. However, it might be interesting to focus on each cognitive ability to understand whether their impairment affects all mathematical skills or just a specific one.

## Figures and Tables

**Table 1:** Number of studies finding worse performance in the MD group than in the CG for each cognitive domain.

Domain (*N* of Studies)	MD-CG Difference (*N* of Studies)
Processing speed (22)	MD < CG (17/22)
STM verbal (12)	MD < CG (9/12)
STM visuospatial (4)	MD < CG (1/4)
LTM verbal (2)	MD < CG (1/2)
Attention (9)	MD < CG (9/9)
WM verbal (21)	MD < CG (16/21)
WM visuospatial (14)	MD < CG (9/14)
Inhibition (8)	MD < CG (6/8)
Cognitive Flexibility (7)	MD < CG (4/7)
Phonological processing and awareness (4)	MD < CG (2/4)

MD: Group with Mathematical Difficulties; CG: Control Group; STM: Short-Term Memory; LTM; Long-Term Memory; WM: Working Memory.

## Supplementary Materials

The following are available online at https://www.mdpi.com/article/10.3390/brainsci12020239/s1, Table S1: Tools, domains, and criteria used to define the group with MD in included studies.

## Appendix A

**Table A1:** Characteristics of the cross-sectional studies.

Author, Year of Publication	Sample Groups (*N* ^1^, Gender, Mean Age)	Intelligence Assesment	Screening Measures for Mathematics (Test) and Criteria for MD ^2^ Group	Cognitive Domain	Task	Outcome
Webster, 1980 [55]	MD-2 = 26 11.4 (0.26) MD-1 = 24 11.39 (0.29) CG ^3^ = 26 11.53 (0.25)	PPVT ^4^	WRAT ^5^ MD-2 (severe) = WRAT < 2 sd MD-1 (mildly) = WRAT ≤ 1 sd	- Verbal STM ^6^	- Letters and Digits Forward Span	Verbal STM MD-2 < CG MD-1 = CG
Geary et al., 1999 [76]	MD = 15 (8 M ^7^; 7 F ^8^) 6.92 CG = 35 (13 M; 22 F) 6.75	WISC ^9^ III OR Stanford–Binet Intelligence Scale	WIAT ^10^ MD = WIAT < 30°	1. Verbal STM 2. Verbal WM ^11^	1. Digit Forward Span (WISC III) 2. Digit Backward Span (WISC III)	1. Verbal STM MD = CG 2. Verbal WM MD < CG
Keeler & Swanson, 2001 [56]	Study #2 MD = 20 (13 M; 7 F) 11.78 (0.47) CG = 18 (11 M; 7 F) 11.65 (0.34)	Raven Progressive Matrices	WJ ^12^ (calculation subtest) MD = WJ < 25° (90 standard score)	1. Verbal WM 2. Visuospatial WM	1. Digit Sentence Span task 2. Mapping and Directions task	1. Verbal WM MD < CG 2. Visuospatial WM MD < CG
Geary et al., 2004 [75]	MD = 58 (32 M; 26 F) CG = 91 (36 M; 55 F)	Stanford–Binet Intelligence Scales	WIAT MD = WIAT < 30°	- Verbal WM	- Counting Span	Verbal WM MD < CG
Censabella & Noël, 2007 [77]	STUDY #1 MD = 20 (10 M; 10 F) 10.48 (0.54) CG = 20 (10 M; 10 F) 10.33 (1.18) STUDY #2 Only Fact retrievals Deficit MD = 12 CG = 12	WISC-III	Arithmetic Skills: standardized pedagogic task (Simonart,1998) + Arithmetic fact retrieval (ad hoc) MD = ICD-10 ^13^ criteria	- Inhibition	- Listening Span test (suppression of irrelevant information) - Color–Word Stroop test (inhibition of prepotent response) - Flanker task (interference control)	- Suppression of irrelevant information: Intrusion errors: #1 MD = CG #2 CG < MD Span score: #1 MD < CG #2 MD = CG - Inhibition of prepotent response (RTs and Accuracy): #1 and #2 MD = CG - Interference Control (RTs and Accuracy): #1 and #2 MD = CG
Cirino et al., 2007 [64]	MD = 51 (33 M; 18 F) 9.55 (0.7) CG = 85 (43 M; 42 F) 9.3 (0.7)	WASI ^14^	WRAT-3 MD = WRAT-3 ≤ 30°	- Attention	- SWAN ^15^ 4th ed. (inattention scale)	Attention: MD < CG
Rousselle & Noël, 2007 [53]	MD = 29 (8 M; 21 F) 7.32 (0.25) CG-MD = 29 (16 M; 13 F) 7.58 (0.23) MD/RD ^16^ = 16 (6 M; 10 F) 7.41 (0.35) CG-MD/RD = 16 (7 M; 9 F) 7.5 (0.25) MD + MD/RD = 45 (14 M; 31 F) CG = 45 (23 M; 22 F)	WISC-III	TEDI-MATH + Transcoding tokens into Arabic numerals. Untimed addition and subtraction test; Timed addition test (ad hoc) MD = Composite Score < 15° + Teacher reports	- Inhibition	- Numerical Stroop task	Inhibition (RTs and Accuracy) MD + MD/RD = CG
Fuchs et al., 2008 [31]	CG = 415 (201 M; 214 F) MD = 132 (63 M; 69 F) MD- Computational and Problem-Solving (MD-CP) = 64 (32 M; 32 F) MD-Computational (MD-C) = 35 (17 M; 18 F) MD-Problem-solving (MD-P) = 33 (14 M; 19 F)	WASI	WJ-III + Computational skills + Problem-solving MD-CP ≤15° on both computational and problem-solving factors MD- C ≤15° on computational skills MD-P ≤ 15° on problem-solving task	1. Processing Speed 2. verbal WM 3. Verbal LTM ^17^ 4. Attention	1. Visual Matching (WJ-III) 2. Listening Recall (WMTB-C ^18^) 2. Numbers Reversed (WJ-III) 3. Retrieval Fluency (WJ-III) 4. SWAN	1. Processing Speed (accuracy): MD < CG 2. Listening Recall: MD < CG 2. Numbers Reversed: MD-P = MD-CP = MD-C MD-C = CG MD-P = MD-CP < CG 3. Verbal LTM: MD-P = MD-CP < MD-C = CG 4. Attention: MD < CG
Raghubar et al., 2009 [65]	#1 MD = 51 (33 M; 18 F) 9.55 (0.7) CG = 85 (43 M; 42 F) 9.30 (0.7) #2 MD = 36 (17 M; 19 F) 9.88 (0.08) CG = 85 (43 M; 42 F) 9.3 (0.7)	WASI	WRAT-3 #1 MD = WRAT-3 < 30° #2 MD = WRAT < 16° LA = WRAT < 30°	- Attention	- SWAN	Attention: #1 MD < CG #2 MD < CG
Chan & Ho, 2010 [60]	MD = 49 9 (0.08) CG = 76 9 (0.08)	RSPM ^19^	Hong Kong Attainment test on Mathematics + Arithmetic subtest Grades 2 or 3: MD ≤ 25° at screening math tasks Grade 4: MD ≤ 20° at screening math tasks	1. Processing Speed 2. Verbal WM 3. Visual WM	1. Visual Matching (WJ-III) 1. Digit Rapid Naming (HKT-SpLD ^20^) 2. Digit Span Backward (WISC) 3. Visual Memory Subtest (TVPS-NM ^21^)	1. Processing Speed MD = CG 2. Verbal WM MD < CG 3. Visual WM MD = CG
Costa et al., 2011 [93]	MD = 14 (6 M; 8 F) 10.18 (1.07) CG = 84 (30 M; 54 F) 10.17 (1.09)	CPM ^22^	TDE ^23^ MD = TDE < 1 sd	1. Processing speed 2. Verbal STM 3. Verbal WM 4. Visuospatial STM 5. Visuospatial WM	1. Simple Reaction Time 2. Digit span Forward (WISC III) 3. Digit Span Backward (WISC III) 4. Corsi Block Forward 5. Corsi Block Backward	All cognitive domains: MD = CG
Passolunghi, 2011 [66]	MD = 18 (7 M; 11 F) 9.6 (5) CG = 18 (10 M; 8 F) 9.7 (5)	PMA ^24^	AC-MT ^25^ MD = AC-MT < 25° + Teacher report	1. Processing speed 2. Verbal STM 3. Verbal WM	1. Symbol Search (WAIS ^26^) 2. Digit span Forward (WISC-R) 2. Word Forward Span task 3. Listening Span task	1. Processing Speed (Accuracy) MD < CG 2. Verbal STM (Digit and Word) MD = CG 3. Verbal WM Correct Recall MD < CG Intrusion Error MD > CG
Passolunghi & Mammarella, 2012 [67]	MD = 35 (15 M; 20 F) 9.96 (0.77) CG = 35 (14 M; 21 F) 9.94 (0.73)	PMA	Standardized Mathematics test (Word problem solving; Amoretti et al., 1994) MD = Math test < 25° + teacher report	1. Spatial WM 2. Visual WM	1. Simple Matrix task 1. Complex Matrix task 2. Simple House Recognition 2. Complex House Recognition	1. Spatial WM MD < CG 2. Visual WM MD = CG
Peng et al., 2012 [57]	MD = 18 (11 M; 7 F) 11 (0.52) CG = 30 (13 M; 17 F) 10.97 (0.36)	RSPM	WRAT 4 (computation) <25° Math Problem-solving (adapted from WISC IV) <15°	1. Verbal STM 2. Attention 3. Inhibition 4. Updating (Verbal WM)	1. Word Span Forward 1. Digit Span Forward (WISC-IV) 2. Dual-task (Complex reading and computation span) 3. Stroop Color–Word 3. Number Inhibition 4. 2-Back task (word and Digit)	1. Verbal STM Word Span: MD = CG Digit Span: MD < CG 2. Dual-Task MD < CG 3. Inhibition Stroop Color–Word: MD = CG Number inhibition: MD < CG 4. Updating MD = CG
Cai et al., 2013 [44]	*N* = 111 (48 M; 63 F) 11.97 CG = 56 MD = 55	RSPM	Standardized mathematics test (Dong, 2011) MD = −2ds at the standardized test + three recent math tests ≤ 20° of the class	1. Processing speed 2. Planning 3. Attention 4. Successive Processing 5. Simultaneous Processing 6. Phonological Loop 7. Central Executive 8. Visuospatial sketchpad	1. Reaction time 2. Matching Numbers; Planned Codes; Planed Connection (CAS ^27^) 3. Expressive attention; number detection; receptive attention (CAS) 4. Word series; sentence repetition; sentence questions (CAS) 5. Non-verbal matrices; verbal spatial relations; figure memory (CAS) 6. Number Span task 6. Sentence Span task 7. Stop-Signal task 7. Flanker task 8. N-Back task 8. Nine-gride graphical spatial location task	1. Processing Speed MD = CG 2. Planning (all tasks): MD < CG 3: Attention (all tasks): MD < CG 4. Successive processing Sentence questions and repetition: MD < CG Word series: MD = CG 5. Simultaneous Processing (all Tasks): MD < CG 6. Phonological Loop MD < CG 7. Central Executive: MD < CG 8. Visuospatial Sketchpad: MD < CG
De Weerdt et al., 2013a [68]	MD = 22 (6 M; 16 F) 9.79 (0.75) CG = 45 (19 M; 26 F) 12.07 (0.86)	Short form WISC-III	Clinical diagnosis + TTR ^28^ + KRT-R ^29^ (mental arithmetic and number knowledge) MD ≤ 10° on test from clinical diagnosis and at least one standardized math test (TTR and/or KTR-R)	- Inhibition	- Go/No Go task (picture, letter or digit modality)	Inhibition: Commission errors: MD = CG RTs (Go trials) MD = CG
De Weerdt et al., 2013b [69]	MD = 22 (6 M; 16 F) 9.79 (0.75) CG = 45 (19 M; 26 F) 12.07 (0.86)	Short form WISC-III	TTR + KRT-R (mental arithmetic and number knowledge) MD ≤ 11° on at least one standardized math test (TTR and/or KTR-R)	1. Verbal STM 2. Verbal WM 3. Visuospatial STM 4. Visuospatial WM	1. Digit Span Forward 1. Word List Forward 2. Digit Span Backward 2. Word List Backward 2. Listening Span task 3. Corsi Block Recall Forward 4. Corsi Block Recall Backward 4. Spatial Span	1. Verbal STM Digit Span: MD < CG Word Span: MD = CG 2. Verbal WM (All tasks): MD < CG 3. Visuospatial STM: MD = CG 4. Visuospatial WM: Block recall: MD = CG Spatial Span: MD < CG
Moura et al., 2013 [70]	MD = 28 (10 M; 18 F) 9.6(1.1)y.o CG = 81 (36 M; 45 F) 9.4 (1.3)	CPM	TDE MD = TDE < 25°	1. Verbal WM 2. Visuospatial WM	1. Digit Span Backward (WISC-III) 2. Corsi Block Backward	1. Verbal WM MD < CG 2. Visuospatial WM MD = CG
Reimann et al., 2013 [80]	MD = 48 (16 M; 33 F) 8.5 (1.6) CG = 783 (399 M; 384 F) 7.11(1.8)	IDS ^30^	IDS MD IDS < 15°	1. Attention 2. Verbal STM 3. Verbal LTM 4. Visuospatial WM	1. Cross-Out (IDS) 2. Digit and Letter Span Forward (IDS) 3. Story Recall (IDS) 4. Shape Recognition (IDS)	1. Attention MD < CG 2. Verbal STM MD = CG 3. Verbal LTM MD = CG 4. Visuospatial WM MD = CG
Szucs et al., 2013 [71]	DD ^31^ = 12 (8 M; 4 F) 9.2 y.o CG = 12 (5 M; 7 F) 9.1 y.o	CPM + WISC-III- short form	MaLT ^32^ + WIAT-II (numerical Operations subtest) DD ≤ 1 ds (<16°) at both math ability tests	1. Switching 2. Inhibition 3. Verbal STM 4. Verbal WM 5. Visuospatial STM 6. Visuospatial WM	1. Trial Making test 2. Animal Stroop; Numerical Magnitude Comparison Stroop task; Physical Size Comparison Stroop task; Stop-Signal task 3. Digit Span Forward (AWMA ^33^) 3. Word Recall (AWMA) 4. Listening Span test (AWMA) 5. Dot Matrix (AWMA) 6. Odd One Out (AWMA)	1. Switching: accuracy: MD = CG RTs: MD < CG 2. Inhibition 3. Verbal STM MD = CG 4. Verbal WM MD = CG 5. Visuospatial STM MD < CG 6. Visuospatial WM MD < CG
Willcutt et al., 2013 [58]	MD = 183 (82 M; 101 F) 11.4 (2.4) CG = 419 (202 M; 217 F) 11.1 (2.2)	WISC-R	PIAT ^34^ and WRAT subtest MD = −1.25 sd at the composite measure of math calculation	1. Processing speed 2. Phonological Awareness 3. Attention 4. Inhibition 5. Shifting 6. Verbal WM	1. Symbol Search (WISC-III); Coding (WISC-R); Colorado Perceptual Speed test; Identical Pictures (Educational Testing Service) 1. RAN (objects, numbers, letters and colors) 2. Phoneme Deletion task; Pig Latin task 3. Gordon Diagnostic System 4. Stop-Signal task; Gordon Diagnostic system; Stroop Color–Word test 5. WCST ^35^ 6. Sentence Span task; Counting Span task	1. Processing Speed (all tasks): MD < CG 2. Phonological Awareness: MD < CG 3. Attention (omission) MD < CG 4. Inhibition (all tasks) MD < CG 5. Shifting: MD < CG 6. Verbal WM: MD < CG
Attout & Majerus, 2015 [95]	DD ^36^ = 16 (6 M; 10 F) 10.04 (1.38) CG = 16 (6 M; 10 F) 9.47 (1.19)	CPM + EVIP ^37^	Clinical diagnosis of DD + TTR = more than 1 year of delay + NUMERICAL + TEDI-MATH + ZAREKI-R = −2 sd	- Verbal WM	- Item Working Memory task -Order Working Memory task	Verbal WM - Item Working Memory task: MD = CG - Order Working Memory Task: MD < CG
Cirino et al., 2015 [54]	*N*#1 = MD = 105 (46 M; 59 F) 7.74 (0.48) CG = 403 (186 M; 217 F) 7.48 (0.32) LowMD = 56 CG = 530 * age as cov	WASI	WRAT-3 #1 MD = WRAT-3 < 25° #2 LowMD = WRAT-3 < 10°	1. Processing speed 2. Phonological Awareness 3. Verbal WM	1. Cross-Out (WJ III) 1. RAN ^38^ (letters and digits, CTOPP ^39^) 2. Elision (CTOPP) 3. Listening Span task; Digit Span Backward; Counting Recall (WMBT-C)	1. Processing Speed (Cross-Out and RAN) MD < CG 2. 3. Verbal WM: MD < CG #1 Average of all cognitive variables: MD < CG
Kroesbergen & Van Dijk, 2015 [78]	*N* = 154 (70 M; 84 F) 8.65 (0.87) CG = 94 MD = 26	If IQ not available, measured with four subtest of the WISC-III	Cito Math test MD = Cito Math test < 15°	- Visuospatial WM	- Dot Matrix; Odd One Out; Spatial Span (AWMA)	Visuospatial WM: MD < CG
Donker et al., 2016 [47]	MD = 31 (7 M; 24 F) 8.95 (1.02) CG = 34 (12 M; 22 F) 8.57 (.75) * groups differed on age	WISC-III Full-version for children assessed in the past two years (37/133) Short version (96/133)	TTR + Math standardized test—problem solving MD = TTR < 1sd + Math standardized test < 25°	- Processing speed	- RAN Alphanumeric (Digits and letters) - RAN Non-Alphanumeric (colors and pictures)	Processing Speed: Alphanumeric: MD = CG Non-alphanumeric: MD < CG
Kuhn et al., 2016 [79]	DD = 33 (6 M; 27 F) 8.78 (.96) CG = 40 (17 M; 23 F) 8.95 (0.63)	WISC-IV	ZAREKI-R ^40^ DD = ZAREKI-R ≤ 80 (raw score)	1. Processing speed 2. Attention 3. Flexibility 4. Verbal WM 5. Visual WM	1. Choice Reaction Time task 2. FBB-ADHS (inattention Scale) 2. Alertness; Sustained Attention (KITAP) 3. Flexibility (KITAP) 4. Verbal Span task 5. Visual Matrix task	1. Processing Speed: DD = CG 2. Attention -FBB-ADHS: MD = CG -Alertness: MD = CG -Sustained Attention (errors): DD > CG (omission): DD = CG 3. Flexibility (accuracy and RTs) DD = CG 4. Verbal WM: DD < CG 5. Visual WM: DD < CG
Slot et al., 2016 [61]	MD = 26 8.89 (0.99) CG = 32 8.61 (0.73)	WISC- III	TTR + Cito Math test MD = TTR < 1 sd + Cito Math test ≤ 25°	1. Processing Speed 2. Phonological Awareness 3. Verbal STM 4. Visuospatial WM	1. RAN (letters, digits, pictures and colors) 2. Phoneme Deletion; Phoneme Manipulation (FAT ^41^) 3. Digit Span Forward; Word Recall (AWMA) 4. Dot Matrix; Spatial Span; Odd One Out (AWMA)	1. Processing Speed: MD = CG 2. Phonological Awareness: MD = CG 3. Verbal STM: MD = CG 4. Visuospatial WM: MD = CG
Lafay & St-Pierre, 2017 [59]	DD = 24 (4 M; 20 F) 9.02(0.36) CG = 37 (16 M; 21 F) 8.9 (0.32) * Groups differ for gender and SES	CPM (Raw score)	ZAREKI-R DD = ZAREKI-R= −1.5 ds	1. Processing speed 2. Verbal STM 3. Verbal WM 4. Visuospatial STM 5. Visuospatial WM	1. Coding (WISC-IV) 2. Digit Span Forward (ZAREKI-R) 3. Digit Span Backward (ZAREKI-R) 4. Corsi Block Forward 5. Corsi Block backward	1. Processing Speed (accuracy) DD < CG 2. Verbal STM DD = CG 3. Verbal WM DD < CG 4. Visuospatial STM DD = CG 5. Visuospatial WM DD = CG
Lambert & Spinath, 2018 [72]	#1 *N* = 229 (119 M; 110 F) 9.18 (1.09) MD = 43 CG = 186 #2 *N* = 120 (50 M; 70 F) 9.16 (0.72) MD = 27 CG = 60	2nd grade CFT 1 3rd–4th grade CFT 20-R + CPM * IQ as cov	DEMAT 1 MD ≤ 25° on DEMAT 1	- Processing Speed	- Numerosity Processing Speed	Processing Speed: MD < CG
Mammarella et al., 2018 [73]	MD = 24 (14 M; 10 F) 9.78 (0.61) CG = 24 (10 M; 14 F) 9.8 (0.58)	WISC-IV (Block Design and Vocabulary subtest)	AC-MT + AC-FL ^42^ (Math Fluency) MD ≤ 10° in at least two mathematical tasks ≤16° at the total mathematical score LA ≤ 20° in at least two mathematical tasks ≤30° at the total mathematical score	- Visuospatial WM	- Visual Working Memory task (houses and balloons) - Spatial-Simultaneous Matrices task (grid, no grid) - Spatial-Sequential Matrices task (grid, no grid)	Visuospatial WM (al tasks): MD < CG
McDonald & Berg, 2018 [74]	MD = 20 (7 M; 13 F) 9.7 (0.56) CG = 20 (11 M; 9 F) 9.8 (0.56) CG-Y = 20 (11 M; 9 F) 7.83 (0.63)	CPM	WIAT- II MD = WIAT-II < 25°	1. Inhibition 2. Shifting 3. Verbal WM 4. Visuospatial WM	1. Quantity–Digits Inhibition task. 1. Color–Word Stroop task 2. Making Trials task (letters and digits) 3. Auditory Digit Sequence; Semantic Categorization (S-CPT ^43^) 4. Visual Matrix task; Mapping and Direction task (S-CPT)	1. Inhibition -Quantity–Digits: MD < CG -Color–Word: MD = CG 2. Shifting (all task) MD < CG 3. Verbal WM (al tasks) MD = CG 4. Visuospatial WM MD < CG

^1^ *N* = Sample; ^2^ MD = Mathematical Difficulties; ^3^ CG = Control Group; ^4^ PPVT = Peabody Picture–Vocabulary Test. ^5^ WRAT = Wide Range Achievement Test; ^6^ STM = Short-Term Memory; ^7^ M = Male; ^8^ F = Female; ^9^ WISC = Wechsler Intelligence Scale for Children; ^10^ WIAT = Weschler Individual Achievement Test (Mathematics Reasoning subtest).; ^11^ WM = Working Memory; ^12^ WJ-III = Woodcock–Johnson Test of Achievement; ^13^ ICD-10 = International Classification of Disease, 10. ^14^ WASI = Wechsler Abbreviated Scale of Intelligence; ^15^ SWAN = Strengths and Weaknesses of ADHD and Normal Behavior; ^16^ MD/RD = Mathematical and Reading Disabilities; ^17^ LTM = Long-Term Memory; ^18^ WMTB-C = Working Memory Test Battery for Children. Pickering & Gathercole, 2001; ^19^ RSPM = Raven’s Standard Progressive Matrices; ^20^ HKT-SpLD = Hong Kong Test of Specific Learning Difficulties; ^21^ TVPS-NM. Test of Visual–Perceptual Skills (non-motor); ^22^ CPM = Raven’s Colored Progressive Matrices; ^23^ TDE = Teste de Desempenho Escolar; ^24^ PMA = Primary Mental Abilities; ^25^ AC-MT = Assessment of Arithmetic Calculation (Test di valutazione delle Abilità di Calcolo- gruppo MT); ^26^ WAIS = Wechsler Adult Intelligence Scale; ^27^ CAS = Cognitive Assessment System; ^28^ TTR = Tempo Test Rekenen (Tempo Test Arithmetic); ^29^ KRT-R = Kortrijkse Rekentest Revisie (Kortrijk Arithmetic test Revision); ^30^ IDS = Intelligence Development Scales; ^31^ DD = Developmental Dyscalculia; ^32^ MaLT = Mathematics Assessment for Learning and Teaching test. *5*; ^33^ AWMA = Automated Working Memory Assessment; ^34^ PIAT = Peabody Individual Achievement Test; ^35^ WCST = Wisconsin Card-Sorting Test; ^36^ DD = Developmental Dyscalculia; ^37^ EVIP = Echelle de Vocabulaire en Images Peabody (French adaptation of the Peabody Picture–Vocabulary Test). Dunn et al., 1993; ^38^ RAN = Rapid Automatized Naming; ^39^ CTOOP = Comprehensive Test of Phonological Processing; ^40^ ZAREKI-R = Neuropsychologische Testbatterie für Zahlenverarbeitung und Rechnen bei Kindern - Revidierte Fassung.; ^41^ FAT = Fonemische Analyse Test; ^42^ AC-FL = Nuove prove di fluenza matematica (Mathematics Fluency); ^43^ S-CPT = Swanson Cognitive Processing Test. * Groups significantly differ on the variable.

**Table A2:** Characteristics of the longitudinal studies.

Author, Year of Publication	Sample Groups (*N* ^1^, Gender, Mean Age)	Intelligence Assessment	Screening Measures For Mathematics (Test) and Criteria for MD ^2^ Group	Cognitive Domain	Task	Follow-Up	Outcome
Geary et al., 2000 [84]	T1 MD = 12 (7 M ^3^; 5 F ^4^) 6.92 CG ^5^ = 26 (12 M; 14 F) 6.75	T2 WISC ^6^- III (vocabulary and Matrix reasoning subtests) or Stanford–Binet Intelligence Scale. * IQ as Cov	T2 and T4 WIAT ^7^	T1 and T3 1. Verbal STM ^8^ 2. Verbal WM ^9^	1. Digit Span Forward (WISC III) 2. Digit Span Backward (WISC III)	T1-1st grade Fall T2-1st grade Spring T3-2nd grade Fall T4-2nd grade Spring	1. Verbal STM: MD = CG 2. Verbal WM MD = CG
Passolunghi & Siegel, 2004 [91]	T1 MD = 22 (14 M; 13 F) 10.4 CG = 27 (11 M; 11 F) 10.4	T1 PMA ^10^	T1 and T2 Standard math test (Amoretti et al., 1994) T2 WRAT ^11^-3 (arithmetic subtest)	T2 1. Verbal STM 2. Simple Verbal WM 3. Complex WM	1. Digit/Word Span Forward 2. Digit/Word Span Backward 3. Listening Span task; Listening Completion task; Counting Span task	T1-4th grade T2-5th grade	1. Verbal STM MD = CG 2. Simple Verbal WM - Word: MD = CG - Digit: MD < CG 3. Complex WM - Listening Span and Completion task (correct recall; order): MD < CG (intrusion error): MD > CG - Counting Span task (correct Recall; order): MD < CG (intrusion): MD > CG
Geary et al., 2007 [88]	MD = 15 T1 = 6.08 ± 0.34 CG = 46 (29 M; 17 F) T1 = 6.34 ± 0.34	T1-CPM ^12^ T2-WASI ^13^ * IQ as cov	T1 and T2 WIAT-II-A	T2 1. Central Executive 2. Visuospatial Sketchpad 3. Phonological Loop T3 4. Processing Speed	1. Listening Recall; Counting Recall; Digit Recall Backward (WMTB-C ^14^) 2. Block Recall; Mazes Memory task (WMTB-C) 3. Digit Recall; Word List Recall; Non-Word List Recall; Word List Matching task (WMTB-C) 4. RAN ^15^ (digit and letters)	T1-K ^16^ T2-1st grade Fall T3-1st grade Spring	1. Central Executive: MD < CG 2. Visuospatial Sketchpad: MD < CG 3. Phonological Loop: MD < CG 4. Processing Speed (composite score) MD < CG
Mazzocco & Kover, 2007 [86]	T1 *N* = 178 (85 M; 93) M = 6.83 (0.30) F = 6.71 (0.30) MD = 10 CG = 168	T2 WASI	T1; T2 and T3 TEMA ^17^ + WJ-R ^18^ MD = TEMA < 10° in all assessment	T1; T2 and T3 - Executive Functions	CNT ^19^ 1. Baseline task: naming items by color or shape 2. One attribute switching task (alternating between naming color or shape based on one attribute) 3.Two-attribute switching task (alternating between naming color or shape based on two attribute)	T1-1st grade T2-3rd grade T3-5th grade	1. Baseline task Self-corrections MD > CG Efficiency (speed–accuracy trade-off): MD < CG 2. One attribute task MD = CG 3. Two-attributes task MD group omitted from analysis (1/10 child completed the task on the 1st assessment)
Murphy et al., 2007 [38]	T1 MD-10° = 22 (15 M; 7 F) 5.77 (0.49) MD-25° = 42 (16 M; 26 F) 5.86 (0.32) CG = 146 (72 M; 74 F) 5.77 (0.32)	T4 WASI	From T1 to T4: TEMA-2	All grades 1. Processing Speed T2 and T4 2. WM T4 3. Reactive flexibility	1. RAN (digit and colors) 2. One attribute (CNT) 3. Two attributes (CNT)	T1 = K T2 = 1st grade T3 = 2nd grade T4 = 3rd grade	1. Processing Speed —(Digit) From 1st to 3rd grade MD10° < CG 1st and 2nd grade MD25° < CG 3rd grade MD25° = CG - (Colors) K to 2nd grade MD10° < CG 3rd grade MD10° = CG K and 1st grade MD25°< CG 2nd and 3rd grade MD25° = CG 2. WM 1st grade Efficiency: MD10° < CG 3rd grade MD10° < CG MD25° < CG 3. Flexibility: 4th grade MD10° < CG MD25°< CG
Geary et al., 2008 [87]	T1 MD = 19 (9 M; 10 F) 7.83 ±. 25 CG = 50 (24; 26 F) 7.25 ± 0.34	K CPM T1 WISC	T1 and T2 WIAT-II- A	T1 and T2 1. Processing Speed T1 2. Central Executive 3. Phonological Loop; 4. Visuospatial Sketchpad)	1. RAN (digit and letters) 2. Listening Recall; Counting Recall; Digit Recall Backward (WMTB-C) 3. Digit Recall; Word List Recall; Non-Word List Recall; Word List Matching task (WMTB-C) 4. Block Recall; Mazes Memory task (WMTB-C)	T0-K T1-1st grade T2-2nd grade	1. Processing speed: MD < CG 2. Central Executive: MD < CG 3. Phonological Loop: MD < CG 4. Visuospatial Sketchpad: MD = CG
Geary et al., 2012a [89]	T1 = 7 MD = 16 (6 M; 10 F) CG = 132 (68 M; 64 F)	K -CPM T1 -WASI	T2 to T6 -Addition strategy choices; -Number sets; -Number line estimation	T2 to T6 1. Processing Speed T2 and T6 2. Central Executive 3. Phonological Loop 4. Visuospatial Sketchpad 5. Attention	1. RAN (digit and letters) 2. Listening Recall; Counting Recall; Digit Recall Backward (WMTB-C) 3. Digit Recall; Word List Recall; Non-Word List Recall; Word List Matching task (WMTB-C) 4. Block Recall; Mazes Memory task (WMTB-C) 5. SWAN	T1-K T2-1st grade T3-2nd grade T4-3rd grade T5-4th grade T6-5th grade	1. Processing speed MD < CG 2. Central Executive MD < CG 3. Phonological Loop: MD < CG 4. Visual Sketchpad: MD < CG 5. Attention MD < CG
Geary et al., 2012b [90]	MD = 15 (10 M; 5 F) CG = 101 (52 M 49 F)	T1 - CPM T2 - WASI * IQ as cov	From T1 to T4 WIAT-II-A From T3 to T5 - Fact Retrieval (Choice task & Forced Retrieval task)	From T2 to T5 1. Processing Speed T2 2. Central Executive 3. Phonological Loop 4. Visuospatial Sketchpad	1. RAN (digit and letter) 2. Listening Recall; Counting Recall; Digit Recall Backward (WMTB-C) 3. Digit Recall; Word List Recall; Non-Word List Recall; Word List Matching task (WMTB-C) 4. Block Recall; Mazes Memory task (WMTB-C)	T1-K T2-1st grade T3-2nd grade T4-3rd grade T5-4th grade	1. Processing Speed: MD < CG 2. Central Executive: MD < CG 3. Phonological Loop: MD < CG 4. Visuospatial Sketchpad: MD < CG
Mazzocco & Grimm, 2013 [92]	T1 = 249 (120 M; 129 F) T4 = 213 T9 = 161	IQ > 80	All grades TEMA-2 + WJ-R MD = WJ-R < 10°	All grades - Processing speed	- RAN Alphanumeric (letter and digit) - RAN Non-Alphanumeric (colors)	T1-K T2-1st grade T3-2nd grade T4-3rd grade T5-4th grade T6-5th grade T7-6th grade T8-7th grade T9-8th grade	Processing Speed: K (letters, digits, colors) MD < CG 8th grade: (letters and colors): MD < CG (Digits) MD = CG
Cowan & Powell, 2014 [81]	T2 *N* = 199 (99 M; 100 F) 8.92 ± 0.42 MD = 11 (2 M; 7 F) CG = 166 (91 M; 75 F)	T2 CPM * MD < CG	T2 -WIAT-II T1 and T2 (different task, same domain) - Numerical factors -Arithmetic Skills	T1 and T2 1. Processing Speed (visual: T1; Verbal T2) T1 2. Central Executive 3. Phonological Loop 4. Visuospatial Sketchpad	1. Symbol Matching (WISC) 1. Pair Cancellation (WJ III) 1. RAN (letters) 2. Listening Recall (WMTB-C) 3. Word List Recall (WMTB-C) 4. Block Recall; Mazes Memory task (WMTB-C)	T1-1st grade T2-3rd grade	1. Processing Speed (all task): MD < CG 2. Central Executive: MD < CG 3. Phonological Loop: MD < CG 4. Visuospatial Sketchpad: MD = CG
Wong & Chan, 2019 [82]	MD = 79 (45 M; 34 F) 7 CG = 466	T1 CPM * IQ as cov	All grades LAMK-3.0	All grades 1. Verbal WM 2. Visuospatial WM	1. Digit Span Backward 2. Corsi Block Backward	T1 = 1st grade T2 = 2nd grade	1. Verbal WM: MD < CG 2. Visuospatial WM MD = CG
Chu et al., 2019 [62]	MD = 14 Recovered MD = 23 CG = 35	T3 WPPSI ^20^-III * MD < CG	T1 and T2 TEMA T3 WIAT-II (Numerical Operations)	T1 and T2 - Conflict Executive Function scale (Beck et al., 2011)	Inhibition and shifting	T1-K 1st year T2-K 2nd year T3-1st grade	Inhibition and shifting: MD < CG (MD recovered = CG)
Chan & Wong, 2020 [83]	T5 *N* = 101 (56 M; 45 F) 12.08 (±0.33) MD = 14 (7 M; 7 F) 12.17 (±0.33) CG = 87	T2-RSPM (short form, series A–C)	T3, T4 and T5 LAMK	T1 -Verbal WM	- Digit Span Backward	T1 = K T2 = 1st grade T3 = 2nd grade T4 = 2nd grade T5 = 6th grade	Verbal WM MD = CG
Zhang et al., 2020 [63]	*N* = 1471 MD = 6.14 LA = 6.12 CG = 6.17	T6- CPM (short form)	T3 and T7 BAT ^21^	T1 and T2 1. Phonological awareness T2 2. Processing speed	1. Initial Phoneme Identification 2. RAN (objects)	T1 = K fall T2 = K spring T3 = 1st grade fall T4 = 1st grade spring T5 = 2nd grade spring T6 = 3rd grade spring T7 = 4th	1. Phonological Awareness: MD = CG 2. Processing Speed: MD < CG
Träff et al., 2020 [85]	T4 MD = 27 (14 M; 13 F) CG = 81 (51 M; 20 F) 12.95 (±0.27)	T1 to T4 Matrix Reasoning (WISC-IV)	T5 Standardized mathematics test (Swedish National Agency for Education) + overall math accomplishment MD = grade *E* on both criteria	T1 to T4 1. Verbal WM 2. Processing Speed	1. Word Sequence with Interference 2. RAN (Time Color)	T1 = K T2 = 1st grade T3 = 2nd grade T4 = 3rd grade T5 = 6th grade	1. Verbal WM MD = CG 2. Processing Speed MD = CG

^1^ *N* = Sample; ^2^ MD = Mathematical Difficulties; ^3^ M = Male; ^4^ F = Female; ^5^ CG = Control Group; ^6^ WISC = Wechsler Intelligence Scale for Children; ^7^ WIAT = Weschler Individual Achievement Test (Mathematics Reasoning subtest); ^8^ STM = Short-Term Memory; ^9^ WM = Working Memory; ^10^ PMA = Primary Mental Abilities; ^11^ WRAT = Wide Range Achievement Test; ^12^ CPM = Raven’s Colored Progressive Matrices; ^13^ WASI = Wechsler Abbreviated Scale of Intelligence; ^14^ WMTB-C = Working Memory Test Battery for Children; ^15^ RAN = Rapid Automatized Naming; ^16^ K = Kindergarten; ^17^ TEMA = Test of Early Mathematics Ability; ^18^ WJ-R = Woodcock–Johnson Test of Achievement—Revised; ^19^ CNT = Contingency Naming Test; ^20^ WPPSI = Wechsler Preschool and Primary Scale of Intelligence; ^21^ BAT = Basic Arithmetic Test. * groups significantly differ on the variable.
