# Supplementary material for: Domain-General Cognitive Skills in Children with Mathematical Difficulties and Dyscalculia: A Systematic Review of the Literature
Source: Brain Sci. 2022 Feb 10;12(2):239. doi: 10.3390/brainsci12020239 (PMC8870543; doi:10.3390/brainsci12020239)
Supplement: Supplementary file 1 [file brainsci-12-00239-s001.zip › brainsci-1524929-supplementary.pdf]

Table S1. Tools, domains, and criteria used to define the group with MD in included studies.

Math Achievement Test	Domains	Criteria for MD	Study
AC-MT[i] 11-14	Written calculation (+; -; x; :); number knowledge (number magnitude; numerical syntax comprehension)	< 25°	Passolunghi., 2011
		< 10°	Mammarella et al., 2018[b]
AC-FL[ii]	Math Fluency (+; -; x)	<10°	Mammarella et al., 2018[b]
DEMAT[iii]	arithmetic, geometry, numerical sizes	< 25°	Lambert & Spinath, 2018
IDS[iv]	Arithmetical skills (counting; ordinal number; magnitude; invariance; knowledge of Arabic numbers; one step arithmetic word problem)	< 15°	Reimman et al., 2013
KTR-R[v]	Mental arithmetic + number knowledge	< 10°	De Weerdt et al., 2012a[b]
		< 10°	De Weerdt et al., 2012b[b]
MaLT[vi]	All area of math curriculum	< 16°	Szucs et al., 2013[a]
TEMA-2[vii]	Quantities; Counting; comparison; informal arithmetic	< 10° in at least three assessment	Mazzocco & Kover, 2007
		< 25° (or < 10°) in at least two assessment	Murphy et al., 2007
		< 25° at both preschool years	Chu et al., 2019[a]
TDE[viii]	Arithmetic subtest: simple word problems and computational skills (+; -; x; :)	-1 sd	Costa et al., 2011
		< 25°	Moura et al., 2013
TTR[ix]	Arithmetic number facts problems (+; -; x; : and mixed)	< 10°	De Weerdt et al., 2013a[b];
		< 10°	De Weerdt et al., 2013b[b];
		1 year delayed	Attout & Maiereus, 2015[c]
		- 1 sd	Donker et al., 2016[a]
		-1 sd	Slot et al., 2016[a]
WIAT[x]	Mathematics reasoning- basic arithmetic skills (counting; subtraction; reading numbers; time telling)	< 30°	Geary et al., 1999
		< 30°	Geary et al., 2004
		< 25° on 1st grade	Chu et al., 2019[a]
		< 35° in all assessment	Geary et al., 2000

		< 15° in all assessment	Geary et al., 2007
		< 11° in all assessment	Geary et al., 2008
		< 11° in all assessment	Cowan & Powell, 2014 (+ fluency task)
	Numerical Operations Subtest (counting; reading numbers; written calculation for +: -; x; :)	< 16°	Szucs et al., 2013[a]
		< 25°	McDonald & Berg, 2018
WJ-III[xi]	Calculation subtest	< 15°	Fuchs et al., 2008 (+ not validated tests)
		< 25°	Keeler & Swanson
		< 11° in half assessment	Mazzocco & Grimm, 2013
WRAT[xii]	Computational skills (+; -; x; :)	< 25°	Keeler & Swanson, 2001
		< 25°	Peng et al., 2012[a]
		<30° in both years	Passolunghi & Siegel, 2004[a]
	Identification of numbers; counting; number comparison; computational skills (+; -; x; :) Identification of numbers; counting; number comparison; computational skills (+; -; x; :)	< 16°	Raghubar et al., 2009
		<30°	Cirino et al., 2007
		<25°	Cirino et al., 2015
		-1 or 1.5 sd - 2 sd	Webster, 1980
ZAREKI-R[xiii]	Number processing (counting; transcoding; comparison); and calculation (mental; problem solving)	Raw score < 80	Kuhn et al., 2016
		- 1.5 sd	Lafay et al., 2017
Local math standardized test	Standardized Math Test (Amoretti et al., 1994)	< 25°	Passolunghi & Mammarella, 2012
		< 30° in all assessment	Passolunghi & Siegel, 2004[a]
	Standardized math test (Dong, 2011) number; algebra; space and geometry; statistics and probability (+ < 20° on the last three academic exams)	< 20°	Cai et al., 2013
	Standardized pedagogic task (Simonart,1998)- Computational skills (+; - ; x; :)	2 years of delay	Censabella & Noel, 2007 (study #1)

	Cito Math test (Math problem solving; Janssen et al., 2010)	< 15°	Kroesbergen & Van Dijk, 2015
		< 25°	Donker et al., 2016[a]
		< 25°	Slot et al., 2016[a]
	Swedish National Agency for Education (arithmetic; measurement; geometry; fractions; algebra; probability and statics)	Grade *E*	Träff et al., 2020
	LAMK[xiv]	< 25°	Wong & Chan, 2019
		< 25° in both assessment	Chan & Wong, 2019
Math Problem Solving	Adapted from *Arithmetical Reasoning* subtest (WISC-IV)	< 15°	Peng et al., 2012[a]
Composite score	Tedi-Math[xv] (writing numbers and comparison) + computational skills (*ad hoc)*	< 15°	Rousselle & Noël, 2007
	PIAT[xvi] + WRAT-R	-1.25 sd	Willcutt et al., 2013
	Hong Kong Attainment test on Mathematics (arithmetic; measure; shape and space; data handling) + computational skills (+; -; x); Number sense; Number facts; place value	< 25° in 2nd grade<br><br><20° in 3rd and 4th grades	Chan & Ho, 2010
Growth curve		WIAT	Geary et al., 2012a; Geary et al., 2012b
		BAT[xvii]	Zhang et al., 2020

[a]MD group defined by scores under cut-off on both test

[b]MD group defined by scores under cut-off on at least one test

[c]MD group defined by both one-year delay at TTR and scores below 2 sd on at least two subtests of standardized arithmetical tests (ZAKERI-R; Tedi-Math; Numerical)

---

[i]AC-MT= Assessment of Arithmetic Calculation (Test di valutazione delle Abilità di Calcolo- gruppo MT). *Cornoldi et al., 2002*
[ii] AC-FL. Nuove prove di fluenza matematica (Mathematics Fluency). *Caviola et al., 2016*
[iii] DEMAT= Deutscher Mathematiktest [German Test for mathematical achievement]. *Krajewski et al., 2004*
[iv] IDS= Intelligence and Development Scales. *Grob et al., 2009*
[v] KRT-R= Kortrijkse Rekentest Revisie (Kortrijk Arithmetic test Revision). *Baudonck et al., 2006*
[vi] MaLT= Mathematics Assessment for Learning and Teaching test. *Williams, 2005*
[vii] TEMA= Test of Early Mathematics Ability.
[viii] TDE= Teste de Desempenho Escolar. *Stein, 1994*
[ix] TTR= Tempo Test Rekenen (Tempo Test Arithmetic). *De Vos, 1992*
[x] WIAT= Weschler Individual Achievement Test (Mathematics Reasoning subtest). *Weschler, 1992*
[xi] WJ -III= Woodcock-Johnson Test of Achievement
[xii] WRAT= Wide Range Achievement Test
[xiii]ZAREKI-R=Neuropsychologische Testbatterie für Zahlenverarbeitung und Rechnen bei Kindern – Revidierte Fassung. *Von Aster et al., 2006*
[xiv] LAMK: Learning and achievement Mathematical Kit. *Hong Kong Education Bureau, 2015*
[xv] Tedi Math= Test Diagnostique des Compétences de Base en Mathématiques. *Van Nieuwenhoven et al., 2001*
[xvi] PIAT= Peabody Individual Achievement Test.
[xvii] BAT= Basic Arithmetic Test. *Räsänen & Aunola, 2007*
